# *TLR4* Polymorphism, Nasopharyngeal Bacterial Colonization, and the Development of Childhood Asthma: A Prospective Birth-Cohort Study in Finnish Children

**DOI:** 10.3390/genes11070768

**Published:** 2020-07-08

**Authors:** Johanna T. Teräsjärvi, Laura Toivonen, Juho Vuononvirta, Jussi Mertsola, Ville Peltola, Qiushui He

**Affiliations:** 1 Institute of Biomedicine, Research Center for Infections and Immunity, University of Turku, 20520 Turku, Finland; johter@utu.fi (J.T.T.); jusmer@utu.fi (J.M.); 2 Department of Paediatrics and Adolescent Medicine, Turku University Hospital and University of Turku, 20520 Turku, Finland; laura.toivonen@utu.fi (L.T.); vilpel@utu.fi (V.P.); 3 William Harvey Heart Centre, Barts & the London School of Medicine & Dentistry, Queen Mary University of London, London EC1M 6BQ, UK; j.vuononvirta@qmul.ac.uk; 4 Department of Medical Microbiology, Capital Medical University, Beijing 100069, China

**Keywords:** *TLR4*, nasopharyngeal bacterial colonization, asthma, *Moraxella catarrhalis*, *Haemophilus influenzae*, childhood asthma

## Abstract

We aimed to explore the role of TLR4 (rs4986790) polymorphism in the nasopharyngeal (NP) bacterial colonization and its consequent impact on the development of childhood asthma. A semi-quantitative culture of NP swabs was performed on 473 children at 2 months of age and on 213 children at 13 months of age. *TLR4* polymorphism was analyzed for 396 children. Children were followed from birth to the age of 7.5 years and the final outcome was physician-diagnosed asthma. The associations between *TLR4* genotype, bacterial colonization, and asthma were analyzed. Children with TLR4 AG or GG genotype were more often colonized with *Moraxella catarrhalis* at 2 months of age (*p* = 0.009) and *Haemophilus influenzae* at 13 months of age (*p* = 0.018). Children who were colonized with *H. influenzae* at 13 months of age had a significantly higher risk of later development of asthma (*p* = 0.004). *M. catarrhalis* or *H. Influenzae* colonization at 2 months of age or *TLR4* genotype Asp299Gly were not associated with the development of childhood asthma. *TLR4* Asp299Gly polymorphism was associated with an increased risk of colonization of *M. catarrhalis* and *H. influenzae* in children. The colonization with *H. influenzae* at 13 months of age was associated with a higher risk of later development of childhood asthma.

## 1. Introduction

After birth, neonates are rapidly colonized with a variety of microbes. The nasopharyngeal (NP) microbiota is highly dynamic over the first years of life [1]. Some of the NP microbes are potential pathogens, such as *Haemophilus influenzae* and *Moraxella catarrhalis*. These bacteria are associated with susceptibility to respiratory tract infections and increased risk for the later development of asthma, which is the most common chronic disease in children [2,3]. Around 10–20% of children suffer asthma-like symptoms at least once in the childhood, and 4–7% will have pediatric asthma [4,5].

Genetic alterations in the pathogen recognition system of innate immunity may increase risk for recurrent respiratory infections and atopy, which may further cause a predisposition for the development of asthma. Toll-like receptors (TLRs) are crucial components of innate immunity by recognizing pathogen-associated molecular patterns (PAMPs) and damage-associated molecular patterns (DAMPs) [6]. TLR4 is a major signaling receptor for lipopolysaccharides (LPS) of Gram-negative bacteria [6]. The recognition of LPS leads to downstream signaling of TLR4 and induces the release of pro-inflammatory cytokines such as interleukins (IL) [7].

The genetic variations of TLR4 can affect its functions. In *TLR4*, there are two well studied functional polymorphisms, Thr399Ile (rs4986791) and Asp299Gly (rs4986790). These two polymorphisms are co-segregating in many different populations, and in the Finnish population, they are in 100% linkage [8,9]. The polymorphism Asp299Gly impairs TLR4-mediated LPS signaling and results in decreased LPS-stimulated NFκB activity [10]. However, little is known about the role of TLR4 gene variations in the NP bacterial colonization or its consequent impact on the development of childhood asthma. Therefore, we aimed to study the effect of *TLR4* polymorphism (rs4986790) on the NP bacterial colonization and the development of asthma in Finnish children.

## 2. Materials and Methods

### 2.1. Study Design and Sample Collection

This study is part of the prospective observational birth cohort study, called Steps to the Healthy Development and Well-being of Children (STEPS) [11]. The STEPS study was approved by the Ministry of Social Affairs and Health and the Ethics Committee of the Hospital District of Southwest Finland (27 February 2007) and was found ethically acceptable by the Ministry of Social Affairs and Health (STM 1575/2008, STM 1838/2009) and the Ethics Committee of the Hospital District of Southwest Finland (19.2.2008 §63, 15.4.2008 §134, 19.4.2011 §113). The aims of the STEPS study are to investigate the precursors and causes of problems in child health and well-being by using a multidisciplinary approach.

The cohort group consists of all children (*n* = 9936) born in the Hospital District of Southwest Finland between January 2008 to April 2010, and their mothers (*n* = 9811). A total of 1827 children were required in the follow-up group. The children were followed intensively from birth to 2 years of age. After the follow-up, the subjects were followed only with annual questionnaires and no NP samples were taken. All participants or parents of participating children gave their written informed consent.

**Figure 1:**
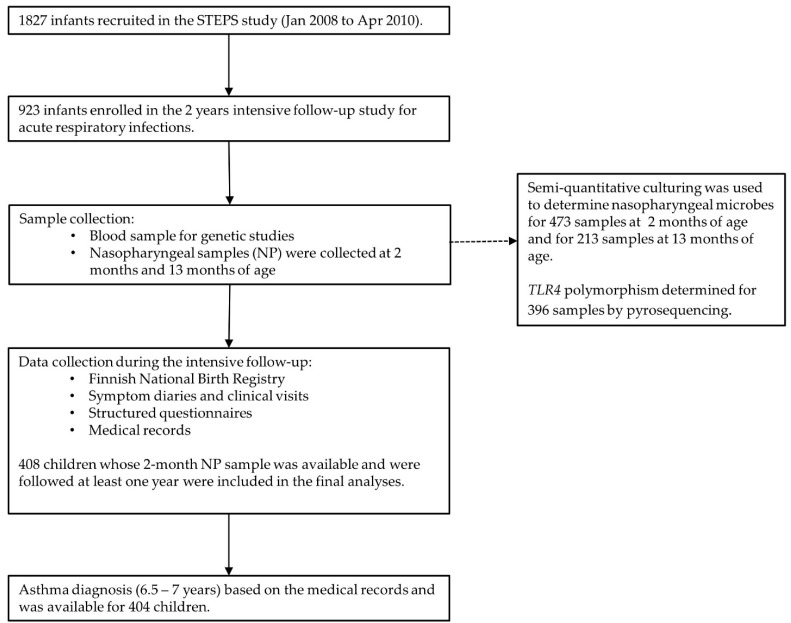
Layout of the study design and sample collection during the follow up.

This study consist of the 473 children from STEP-study follow-up group whose nasopharyngeal sample was taken on the annual visit of study clinic at 2 months of age (Figure 1). Nasopharyngeal samples were collected during prescheduled visits at the age of 2 (average age 2.6 m), 13, and 24 months. Blood samples for genetic studies were collected at the 2-month visit [12].

Children were followed from birth to the age of 7.5 years. Physician-diagnosed asthma was defined as a diagnosis of asthma in the medical records at 6.5–7.5 years of age (later 7 years of age) with or without an electronic prescription of inhaled corticosteroids for asthma at the same age [13]. Children who had been followed at least a year were included in the analyses.

### 2.2. Study Subjects, Genetic Analyses, and Bacterial Culture

Characteristic of the study population is presented in the Table 1. Altogether, 2-month nasopharyngeal bacterial data was available for 473 children and the medical record data of asthma was available for 464 children. The children were followed from birth to the age of 7.5 years and those who were followed at least one year were included in the analyses (*n* = 408). Out of these, 52.9% (*n* = 216) were male and 47.1% (*n* = 192) female.

The majority of children were born vaginally (87.2%) and exclusively or mixed breastfed (88.1%) up to 2 months of age. Only 7.6% of children were fed exclusively with formula. In total, 61.6% of children were first-born and had no older siblings at the moment of birth. The nasopharyngeal follow-up sample at 13 month of age was collected from 213 children and 206 were followed at least one year. The genetic analyses of TLR4 (rs4986790) was performed for 392 (82.9%) children.

DNA was extracted from 200 µL of whole blood by QIAGEN QIAamp DNA Blood Mini Kit 250 (Qiagen, Hilden, Germany) according to the manufacturer’s protocol.

The genotyping of *TLR4* was performed by pyrosequencing (PSQ™96MA Pyrosequencer, Biotage, Uppsala, Sweden), using a PSQ™96 Pyro Gold Q96 reagent kit according to the manufacturer’s protocol. The PCR and sequencing primers used for genotyping of the *TLR4* Asp299Gly (rs4986790) gene was earlier described [14,15,16,17].

The semi-quantitative bacterial culturing method was used for detecting bacteria [12] as described earlier.

### 2.3. Statistical Analysis

The Hardy–Weinberg equilibrium (HWE) was examined for *TLR4* polymorphism by using the Chi-square test. Categorical data were compared by using the Chi-square test or Fisher exact test. The association between risk factors and outcome was analyzed by binary logistic regression analysis and estimated by adjusted odds ratios (OR) with 95% confidence interval (CI) were determined. Potential confounders, namely sex, delivery mode, siblings, time of breast feeding, child’s atopy, recurrent wheezing, and parental asthma, were included in the multivariable models. Two-tailed *p* < 0.05 was considered statistically significant.

The data was analyzed with the use of SPSS software, version 25.0 (IBM Corp. in Armonk, NY, USA).

## 3. Results

### 3.1. TLR4 Polymorphisms and Nasopharyngeal Bacterial Colonization

**Figure 2:**
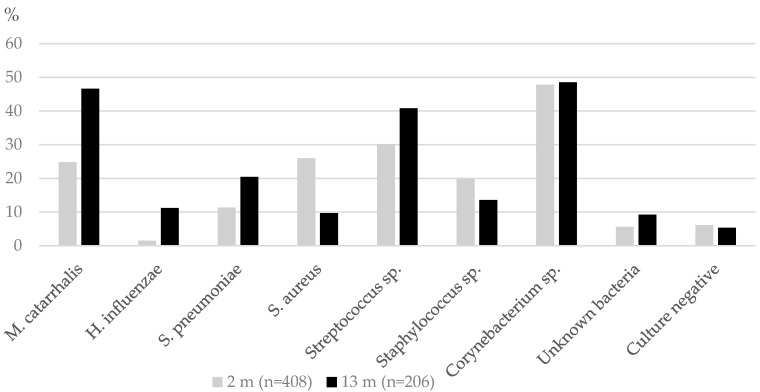
The colonization rates of the studied bacteria at two time points.

The Figure 2 shows the colonization rates of the studied bacteria species at 2 months of age and 13 months. Of studied bacteria, *Sterptococcus pneumoniae*, *M. catarrhalis* and *H. influenzae*, showed increased colonization rates with age, whereas the colonization rate of *Staphylococcus aureus* decreased.

It was found that *TLR4* polymorphism was associated with increased risk for colonization by studied Gram-negative bacteria. Significantly higher colonization rate of *M. catarrhalis* at 2 months of age was found in children with a variant type of *TLR4* than in those with wild type (adjusted odds ratio [aOR], 2.47; 95% CI, 1.25–4.89; *p* = 0.009) (Table 2). In addition, children with a variant type of *TLR4* were more often colonized with *H. influenzae* at 13 months of age (aOR, 4.18; 95%CI, 1.28–13.63; *p* = 0.018) (Table 2). No difference in the colonization rates was found for other studied bacteria between *TLR4* genotypes.

### 3.2. The impact of TLR4 Polymorphism and Nasopharyngeal Bacteria Colonization to Asthma-Susceptibility

Medical record data of asthma were available for 464 children with NP samples at 2 months of age. A total of 37 (7.8%) children had asthma at 7 years of age. Associations of the early NP bacterial colonization with risk of asthma at 7 years of age are shown in Table 3. Early colonization with studied bacteria at 2 months of age were not associated with the development of asthma. However, children colonized with *H. influenzae* at 13 months of age had a significantly higher risk of asthma compared to children not colonized by this bacterial species (aOR, 11.56; 95%CI, 2.14–62.45; *p* = 0.004). *TLR4* variant type was not associated with the development of asthma by age 7 (aOR, 0.20; 95%Cl, 0.23–1.78; *p* = 0.15).

## 4. Discussion

After birth, neonates rapidly acquire a vast number of microbes in different places of the body. Nasopharyngeal bacteria can asymptomatically colonize the nasopharynx of infants and young children but are also associated with the development of respiratory infections during infancy and asthma during childhood. Such nasopharyngeal bacteria include *S. pneumoniae*, *M. catarrhalis*, and *H. influenzae* [2,18]. It is known that the NP bacterial colonization is dynamic over the first years of life. Our finding that the colonization rates of common bacteria in NP samples change within the first year of life in Finnish children is in line with previous studies [19].

In this prospective cohort study, we show that *TLR4* Asp299Gly polymorphism can markedly increase the risk of colonization with two Gram-negative bacteria, *H. influenzae* and *M. catarrhalis*. Children who carry variant type (GA or GG) of *TLR4* were more often colonized by *M. catarrhalis* at 2 months of age and with *H. influenzae* at 13 months of age than children with the wild type (AA) of *TLR4*. The *TLR4* Asp299Gly polymorphism is linked to reduced response to LPS in healthy subjects (16), suggesting that improper response to TLR4 ligand such as LPS can induce an inappropriate immune response. This can increase the risk for repeated colonization of Gram-negative bacteria and further predispose children for recurrent respiratory infections.

In contrast to earlier study with Danish infants [2], we could not find a correlation for the NP colonization with *M. catarrhalis, H. influenza,* or *S. Pneumoniae* being at an increased risk for childhood asthma. The possibility that the number of samples positive for *H. influenzae* at two months of age was too low cannot be excluded. This may also suggest that the genetic variation of TLR4 could not directly be linked to the development of asthma. However, we found that the NP colonization with *H. influenza*e at 13 months of age was associated with an increased risk of developing childhood asthma by age of 7 years. In a recent study using 16S rRNA gene sequencing methods, Teo et al. reported that frequent colonization with Moraxella, Streptococcus, or Haemophilus genera during the first 2 years of life was associated with an increased risk of chronic wheeze [3]. In the current analysis focusing on these species separately, specifically *H. influenzae* was significantly associated with the risk of asthma. Our results build on and expand these earlier studies.

The aim of this study was to explore the possible role of *TLR4* polymorphisms in the development of childhood asthma. For this study, we chose to explore *TLR4* polymorphism rs4986790 because it has been well studied in European populations and there is evidence for its role in respiratory infections and asthma. As mentioned previously, this polymorphism is linked to a reduced response to LPS [18]. With regards to childhood asthma, this SNP seems to lead decreased LPS-induced IL-12(p70) and IL-10 responses and further increase the risk for asthma, especially atopic asthma [20]. In addition, Lee et al. showed, in a study with an experimental asthma model, that the leukotriene B4 receptor-2 (BLT2) linked cascade plays a pivotal role in LPS/signaling for IL-13 synthesis in mast cells and further exacerbating allergic response [7]. This finding provides insight into the mechanism of how bacterial infection can worsen allergic inflammation and increase the susceptibility to chronic infections and asthma.

Contrary to these above-mentioned studies, Douville et al. found that neither *TLR4* SNPs (rs4986790 or rs4986791) influenced immune responses evoked by LPS exposure or RSV infection [21]. In this present study, we could not find any direct associations between studied *TLR4* polymorphism and an asthma risk. However, we observed an indirect association between the studied *TLR4* polymorphism, increased *H. influenzae* colonization and an asthma risk.

Our study has potential limitations. The NP bacteria were analyzed by culture-based methods and only live bacteria were taken into account. The colonization rate of *H. influenzae* at 2 months of age was only 1.6%, and the low detection rate did not give enough statistical power. The number of detected pathogens was not large, especially when several confounders were included in the logistic regression analysis. We did not collect data about the severity of asthma or responsiveness to medication in this study. From a clinical point of view, it would be important to study the effect of *TLR4* polymorphism on the severity of asthma and responsiveness to medication in the future. Yet, our results from this well-defined birth-cohort study should be of interest both to clinicians and researchers.

## 5. Conclusions

*TLR4* Asp299Gly polymorphism was associated with an increased risk of NP colonization with *M. catarrhalis* and *H. influenzae* in children. The colonization with *H. influenzae* at 13 months of age was associated with a higher risk of developing childhood asthma. However, the *TLR4* polymorphism was not directly linked to the increased risk for childhood asthma. Our results warrant further studies on the impact of host TLR4 genetics on the early bacterial colonization and development of asthma in different populations.

## Figures and Tables

**Table 1:** Characteristic of the study cohort.

Characteristic	*n* = 473 (%)
Gender	
Female	221 (46.7)
Male	252 (53.3)
Mode of Delivery	
Vaginal	402 (85)
Caesarean section	67 (14.2)
Missing data	4 (0.8)
Feeding	
Exclusive breast feeding	190 (40.2)
Partial breast feeding	160 (33.8)
Formula	28 (5.9)
Missing data	95 (20.1)
Older siblings	283 (59.8)
Missing data	2 (0.5)
Atopy at 13 months of age	57 (15.1)
Missing data	31 (7.6)
Recurrent Wheezing	48 (12.0)
Missing data	2 (0.5)
Parental asthma	51 (1.6)
Missing data	2 (0.5)
Asthma diagnose (6.5–7.5years)	32 (7.9)
Missing data	4 (1.0)
Genotypes	
*TLR4* (rs4986790) HWE * *p* = 0.54	
AA	326 (83)
AG	64 (16)
GG	2 (1)

Data are presented as numbers (n) of children and percentages (%). A/A indicates wild type and A/G or G/G variant type of *TLR4*. * The Hardy–Weinberg equilibrium (HWE) was examined by Chi-square test. The *p*-value > 0.05 consider as not significant and the studied *TLR4* polymorphism is in Hardy-Weinberg equilibrium in this population.

**Table 2:** Association between *TLR4* gene polymorphism Asp299Gly (rs4986790) and nasopharyngeal bacterial colonization rates at 2 months and 13 months of age.

	*TLR4* Genotype			
**Cultured Bacteria (2 Months)**	**A/A (*n* = 252)**	**A/G or G/G (*n* = 53)**	**aOR (95% Cl) ^†^**	***p* value**
*M. catarrhalis*	55 (21.8)	21 (40.4)	2.47 (1.25–4.89)	0.009 *
*H. influenzae*	5 (2.0)	0 (0)	-	0.99
*S. pneumoniae*	28 (11.1)	8 (15.1)	1.3 (0.52–3.24)	0.58
*S. aureus*	63 (25.0)	13 (24.5)	0.99 (0.5–2.00)	0.99
*Streptococcus sp.*	77 (30.6)	13 (24.5)	0.77 (0.38–1.53)	0.45
*Staphylococcus sp.*	54 (21.4)	6 (11.3)	0.46 (0.19–1.13)	0.10
*Corynebacterium sp.*	121 (48.0)	23 (43.4)	0.84 (0.46–1.54)	0.57
*Neisseria sp.*	3 (1.2)	0 (0.00)	-	0.99
No cultured bacteria	14 (5.6)	3 (5.7)	1.00 (0.27–3.72)	0.99
**Cultured Bacteria (13 months)**	**A/A (*n* = 117)**	**A/G or A/A (*n* = 23)**	**aOR (95% Cl) ^†^**	***p* value**
*M. catarrhalis*	53 (45.3)	13 (56.5)	1.82 (0.69–4.80)	0.23
*H. influenzae*	11 (9.4)	6 (26.1)	4.18 (1.28–13.63)	0.018 *
*S. pneumoniae*	25 (21.4)	2 (8.7)	0.33 (0.07–1.55)	0.16
*S. aureus*	12 (10.3)	1 (4.3)	0.36 (0.04–3.15)	0.36
*Streptococcus sp.*	41 (35.0)	12 (52.2)	2.12 (0.85–5.30)	0.11
*Staphylococcus sp.*	17 (14.5)	4 (17.4)	1.22 (0.36–4.16)	0.75
*Corynebacterium sp.*	56 (47.9)	13 (56.5)	1.58 (0.63–4.00)	0.33
*Neisseria sp.*	5 (4.3)	1 (4.3)	0.96 (0.10–9.25)	0.97
No cultured bacteria	6 (5.1)	1 (4.3)	0.65 (0.07–6.05)	0.71

Data are presented as numbers (n) of children and percentages (%). A/A indicates wild type and A/G or G/G variant type of *TLR4*. aOR: adjusted odds ratio and ^†^ Cl: confidence interval. ^†^ Association between *TLR4* genotype and the risk of bacterial colonization was analyzed using binary logistic regression analysis (confounders: sex, delivery mode, and older siblings). * Two-tailed *p* < 0.05 considered as significant).

**Table 3:** The effect of colonization of nasopharyngeal bacteria at 2 months and 13 months of age on development of asthma by 7 years of age.

**Cultured Bacteria** **(2 Months)**	**Number of Children (*n* = 371)**	**Number of Children with Asthma at 7 Years of Age ^§^ (*n* = 29)**	**aOR (95% Cl) ^‡,†^**	***p*** **Value**
*M. catarrhalis*	Yes, *n* = 91	9 (9.9)	1.04 (0.4–2.73)	0.94
	No, *n* = 280	20 (7.1)	reference	
*H. influenzae*	Yes, *n* = 6	2 (33.3)	2.41 (0.26–22.54)	0.44
	No, *n* = 365	27 (7.4)	reference	
*S. pneumoniae*	Yes, *n* = 40	4 (10)	1.10 (0.27–4.39)	0.90
	No, *n* = 331	25 (7.6)	reference	
*S. aureus*	Yes, *n*=95	9 (9.5)	0.85 (0.32–2.24)	0.74
	No, *n* = 276	20 (7.2)	reference	
**Cultured Bacteria (13 months)**	**Number of Children (*n* = 189)**	**Number of Children with Asthma at 7 years of age ^§^ (*n* = 15)**	**aOR (95% Cl) ^†^**	***p* value**
*M. catarrhalis*	Yes, *n* = 90	9 (10.0)	1.49 (0.39–5.74)	0.56
	No, *n* = 99	6 (6.1)	reference	
*H. influenzae*	Yes, *n* = 22	5 (22.7)	11.56 (2.14–62.45)	0.004 *
	No, *n* = 167	10 (6.0)	reference	
*S. pneumoniae*	Yes, *n* = 39	4 (10)	2.54 (0.51–12.63)	0.25
	No, *n* = 150	11 (7.3)	reference	
*S. aureus*	Yes, *n* = 18	4 (22.2)	4.36 (0.82–23.24)	0.09
	No, *n* = 171	11 (6.4)	reference	

Data are presented as numbers (n) of children and percentages (%). ^‡^ aOR: adjusted odds ratio and ^†^ CI: confidence interval. ^§^ The physician-diagnosis of asthma at 7 years of age was collected from l medical records. Association between bacterial colonization and the risk of asthma was analyzed using binary logistic regression analysis (confounders: sex, delivery mode, siblings, child’s atopy, recurrent wheezing, and parental asthma). * Two-tailed *p* < 0.05 considered as significant.
